# Effect of Heweianshen Decoction on Orexin-A and Cholecystokinin-8 Expression in Rat Models of Insomnia

**DOI:** 10.1155/2016/8034263

**Published:** 2016-09-05

**Authors:** Fagen Li, Shaodan Li, Yi Liu, Ke Cao, Minghui Yang

**Affiliations:** Department of Traditional Chinese Medicine, Chinese People's Liberation Army General Hospital, Beijing 100853, China

## Abstract

*Objective*. To study the effect of Heweianshen decoction (HAD) on orexin-A and cholecystokinin-8 (CCK-8) expression in rat models of insomnia caused by injecting parachlorophenylalanine (PCPA) intraperitoneally.* Methods*. Fifty male Wistar rats were randomly divided into five groups (10 rats in each group): blank group, model group, and low-, medium-, and high-dose HAD-treated groups. A rat model of insomnia was established by injecting intraperitoneally with PCPA (300 mg/kg body weight). Rats were given normal saline (10 mL/kg) or 5.25, 10.5, and 21 g/kg HAD by intragastric administration once a day for 6 days. After that, the rats were sacrificed to collect the hypothalamus for tests, using radioimmunoassay to detect the expression of orexin-A and CCK-8.* Results*. Heweianshen decoction reduced the expression of orexin-A and increased the expression of CCK-8 in the hypothalamus of rat model of insomnia.* Conclusion*. The therapeutic effect of HAD on insomnia is partially attributed to the decreased expression of orexin-A and increased expression of CCK-8.

## 1. Introduction

Insomnia, which is a common problem in psychiatry, is defined as difficulty in initiating, maintaining sleep, or having nonrestorative sleep for at least one month. Insomnia has a high prevalence and causes a lot of harm to people's health. Medications used in the treatment of insomnia include benzodiazepine receptor agonists, nonbenzodiazepine receptor agonists, the selective melatonin receptor agonist ramelteon, and sedating antidepressants [1]. However, these medications have associated risks of side effects or adverse events, and the clinical efficacy remains controversial.

Through long-term clinical practice, we have tested Heweianshen decoction for the treatment of insomnia and achieved satisfactory therapeutic effects [2, 3]. Our previous research has shown that Heweianshen decoction (HAD) has potential for treating insomnia [4]. In present study, we used HAD to intervene with insomnia in rats. By observing the brain neurotransmitters orexin-A and cholecystokinin-8 (CCK-8), we tried to explore the potential therapeutic mechanisms of HAD.

## 2. Materials and Methods 

### 2.1. Animals

Fifty male specific pathogen-free Wistar rats were purchased from Beijing Vital River Laboratory Animal Technology Co. Ltd. [(200 ± 20) g, qualified number SCXK (Beijing) 2014-0003]. Animals were maintained at 23°C ± 1°C and 40%~50% humidity for 12 h in light (7:00~19:00) and 12 h in the dark (19:00~7:00). The experiment was approved by the Animal Ethics Committee of Chinese PLA General Hospital.

### 2.2. Drugs and Reagents

All ingredients of HAD were provided by the Pharmacy of Traditional Medicine of Chinese PLA General Hospital. The ingredients were Banxia (*Pinellia ternata*) 10 g, Yiyiren (Semen Coicis) 15 g, Chen Pi (Tangerine Peel) 15 g, Fuling (*Poria cocos*) 15 g, Shi Chang Pu (*Acorus gramineus*) 10 g, He Huan Pi (*Cortex albiziae*) 10 g, Zaoren (Jujube Benevolence) 15 g, and Ye Jiao Teng (Tuber Fleeceflower Stem) 15 g. Above is the amount of a day for 60 kg body weights adults. PCPA was provided by Shanghai Sigma-Aldrich Trading Co., Ltd. (Shanghai, China). Orexin-A radioimmunoassay kit and CCK-8 radioimmunoassay kit were both purchased from Shanghai Phoenix Pharmaceutical Technology Co., Ltd. (Shanghai, China).

### 2.3. Equipment and Instruments

They are EthoVision XT animal behavior tracking system (Noldus Information Technology Co., Ltd., Gelderland, Netherlands), MP31001 electronic scales (Shunyu Hengping Technology Instrument Co., Ltd., Shanghai, China), and refrigerated high-speed centrifuge (Eppendorf China Ltd., Shanghai, China).

### 2.4. Establishing Rat Models of Insomnia

Six grams of PCPA was weighed and dissolved into 200 mL of normal saline to prepare a 30 mg/mL solution, which was shaken well to allow it to fully dissolve. Rats were injected intraperitoneally with PCPA (300 mg/kg body weight) between 08:00 and 09:00 a.m. once a day for two days to induce insomnia [5]. 75% alcohol was used to disinfect the wound. The rats of blank group were injected intraperitoneally with an equal volume of normal saline. Model rats showed a disorder of circadian rhythms and keep active during the daytime, which were obviously different from the blank group rats [6].

### 2.5. Grouping and Treatment

Animals were divided into the following five groups using the random number table method (10 rats in each group): blank group (10 mL/kg normal saline given by intragastric administration once daily), PCPA model group, and low-, medium-, and high-dose HAD-treated groups. The blank group and PCPA model group were given normal saline by intragastric administration once daily. The dose conversion used for HAD-treated groups was 2.5, 5, and 10 times 60 kg body weights adults dose. The dosage in the low-, medium-, and high-dose groups was 5.25, 10.5, and 21 g·kg^−1^·d^−1^, respectively. All the group of gastric volume was assumed to be 1 mL/100 g in the morning, and treatments lasted for 6 days after model establishment.

### 2.6. Behaviour Observation

EthoVision XT animal behavior tracking system was used to record the video of the behavioral activity of rats for 3 minutes in each group after model establishment but before treatment. The video was recorded again one hour after the last intragastric administration. The walking time and forelimb lifting-up frequency of rats were recorded, respectively.

### 2.7. Radioimmunoassay

On the 6th day after administration, all rats were sacrificed after anesthesia. The hypothalami were dissected, weighed, and fully homogenized in glass homogenate tubes containing 1 mL HCl (1 mol/L) in an ice bath. After homogenization, samples were kept at room temperature for 100 min and centrifuged at 4000 r/min for 20 min at 4°C. 1 mL NaOH (1 mol/L) was added to the supernatant to neutralize the acid. Neutralized samples were centrifuged at 4000 r/min for 10 min and the supernatant (hypothalamus homogenate) was kept at −20°C. The expression of the orexin-A and CCK-8 was determined by radioimmunoassay. All procedures were strictly completed in accordance with radioimmunoassay kit instructions.

### 2.8. Statistical Analysis

The data were expressed as mean ± SD. One-way ANOVA was performed using SPSS 17.0 software (IBM SPSS, Shanghai, China). *P* < 0.05 was used as the threshold for significance.

## 3. Results

### 3.1. Behaviour Observation

After model establishment, the rats lost circadian rhythm and kept active with irritability during the daytime, and the walking time and forelimb lifting-up frequency of PCPA model group and HAD-treated groups were all significantly higher than those of blank group. After treatment, the walking time and forelimb lifting-up frequency of low-, medium-, and high-dose HAD-treated groups were all reduced significantly (*P* < 0.01) (Table 1). The reduction in the high-dose HAD-treated group was most obvious, with a slightly less obvious effect on the medium-dose HAD-treated group and low-dose HAD-treated group.

### 3.2. HAD Affects Orexin-A Expression in Hypothalamus in Rat Model of Insomnia

Compared with the blank group, the level of orexin-A in the PCPA model group was significantly increased (*P* < 0.01). Compared with the PCPA model group, the levels of orexin-A in medium- and high-dose HAD-treated groups were reduced significantly after treatment (*P* < 0.01), and the reduction in the high-dose group was more obvious (Table 2). The level of orexin-A in the low-dose HAD-treated group was lower than that of the PCPA model group, but there was no significant difference (*P* = 0.17).

### 3.3. HAD Affects CCK-8 Expression in Hypothalamus in Rat Model of Insomnia

Compared with the blank group, the level of CCK-8 in the PCPA model group was significantly reduced (*P* < 0.01). Compared with the PCPA model group, the levels of CCK-8 in medium- and high-dose HAD-treated groups were increased significantly after treatment (*P* < 0.01), and the reduction in the high-dose group was more obvious (Table 3). The level of CCK-8 in the low-dose HAD-treated group was lower than that of the PCPA model group, but there was no significant difference (*P* = 0.16).

## 4. Discussion

Insomnia, as one of the most common sleep disorders, seriously affected people's life, work, and health. The pathogenesis of insomnia is complex and still unclear. The treatment methods of modern medicine are limited and the curative effect is not ideal. Through long-term clinical practice under the guidance of traditional Chinese medicine, we have tested Heweianshen decoction for the treatment of insomnia and achieved satisfactory therapeutic effects [2, 3]. However, the curative mechanism is not clear. The present study observes the behavioral activities of the rat model of insomnia and the contents of orexin-A and CCK-8 in hypothalami to explore the action mechanism of Heweianshen decoction treating insomnia.

Orexin is a small-molecule neural polypeptide synthesized and secreted by the lateral hypothalamic area [7, 8]. Existing studies [9] show that orexin is a key factor for regulating sleep-wake cycle, especially for keeping awake. Adamantidis et al. [10] activated the orexin neurons by neural light stimulation technology in the body and found it could facilitate the transformation of slow wave sleep and rapid eye movement sleep (REMS) to waking state. Kiyashchenko et al. [11] detected that the orexin neurons were much more active in wakefulness or REMS than those in quiet sleep. Other studies found that orexin could activate monoamine/cholinergic nerve pathways and facilitate the release of adrenal cortical hormone and norepinephrine and regulate sleep-wake cycle [12, 13]. The latest clinical research of suvorexant [14], orexin receptor antagonist, showed that patients taking suvorexant achieved good effects in a phase III randomized, double-blind, placebo-controlled clinical trial of insomnia treatment. It found that suvorexant could obviously shorten the fall-sleep time and prolong the sleep time of insomnia patients. Above all, orexin has been closely involved in regulating sleep-wake cycle.

CCK is a kind of brain-gut peptide with various biological effects, widely distributed in the central and peripheral nervous system, digestive system, and peripheral blood [15]. CCK-8 is a kind of CCK with highest content in human body, mainly distributed in central nervous system. Research [16–18] showed that CCK participated in a variety of physiological functions and had been closely associated with sleep regulation. Kapás et al. [19] found that CCK could decrease the waking time of rats and rabbits and shorten the latent period of nonrapid eye movement sleep (NREMS) and prolong the time of NREMS, which indicated that CCK could effectively regulate the sleep. Another study discovered that CCK-8S could facilitate the release of *γ*-aminobutyric acid (GABA) in brain, inhibitory neurotransmitter, and prolong the NREMS, which could be blocked by CCK-8S receptor antagonist [20].

This present study indicates that the orexin-A levels in hypothalamus of rat model of insomnia increased significantly (*P* < 0.01) with CCK-8 levels obviously decreased (*P* < 0.01). Following low-, medium-, and high-dose HAD treatments, the orexin-A levels were lower than those following saline treatment, with CCK-8 levels higher. And the effect in high-dose group was most obvious while that of medium-dose group was less obvious and that of low-dose group was not obvious, exhibiting a dose-dependent pattern. The study also indicates that the behavioral activities of rats as walking and forelimb lifting-up in PCPA model group were obviously increased (*P* < 0.01). After treatment, the walking time and forelimb lifting-up frequency of low-, medium-, and high-dose HAD-treated groups were all reduced significantly (*P* < 0.01), which indicates that the sleep of the rat model of insomnia could have been improved with HAD treatment. The results show that HAD could decrease the contents of orexin-A and increase CCK-8 contents in hypothalamus of rat model of insomnia in a dose-dependent pattern, which indicates that the therapeutic effect of HAD on insomnia may be related to the decreased expression of orexin-A and increased expression of CCK-8. How HAD regulates orexin-A and CCK-8 levels and the interaction between these two neurotransmitters needs to be further studied. There could be other factors that play a role in regulating the sleep of rat model of insomnia in this study, for which further research is needed.

## Figures and Tables

**Table 1:** Effect of the Heweianshen decoction on behavioral activities of rat model of insomnia ($\overset{-}{x}\pm s$).

Group	*n*	Walking time (s)		Forelimb lifting-up frequency	
		Before treatment	After treatment	Before treatment	After treatment
Blank group	10	129.25 ± 4.21	130.24 ± 4.53	15.16 ± 1.26	16.36 ± 1.57
PCPA model group	10	159.47 ± 5.46	160.15 ± 5.37^a^	24.32 ± 1.24	25.18 ± 1.72^a^
Low-dose group	10	156.33 ± 6.11	146.38 ± 5.72^*∗*b^	25.12 ± 2.01	21.93 ± 2.18^*∗*b^
Medium-dose group	10	158.21 ± 5.82	139.25 ± 4.58^*∗*b^	24.83 ± 1.67	19.62 ± 1.81^*∗*b^
High-dose group	10	160.15 ± 5.36	135.42 ± 4.61^*∗*b^	24.75 ± 1.76	17.24 ± 2.31^*∗*b^

Notes: blank group and model group were given normal saline 1 mL/100 g; the low-, medium-, and high-dose HAD-treated groups were given Heweianshen decoction of 5.25, 10.5, and 21 g·kg^−1^·d^−1^, respectively. Compared with before treatment, ^*∗*^ *P* < 0.01; compared with the blank group, ^a^ *P* < 0.01; compared with the model group, ^b^ *P* < 0.01.

**Table 2:** Effect of the Heweianshen decoction on orexin-A levels in hypothalamus of rat model of insomnia ($\overset{-}{x}\pm s$).

Group	*n*	Orexin-A (pg/mg)
Blank group	10	19.26 ± 3.38
PCPA model group	10	29.67 ± 5.12^a^
Low-dose group	10	26.71 ± 4.10
Medium-dose group	10	22.03 ± 2.81^b^
High-dose group	10	20.97 ± 3.26^b^

Notes: blank group and model group were given normal saline 1 mL/100 g; the low-, medium-, and high-dose HAD-treated groups were given Heweianshen decoction of 5.25, 10.5, and 21 g·kg^−1^·d^−1^, respectively. Compared with the blank group, ^a^ *P* < 0.01. Compared with the model group, ^b^ *P* < 0.01.

**Table 3:** Effect of the Heweianshen decoction on CCK-8 levels in hypothalamus of rat model of insomnia ($\overset{-}{x}\pm s$).

Group	*n*	CCK-8 (pg/mg)
Blank group	10	1.97 ± 0.54
PCPA model group	10	1.12 ± 0.41^a^
Low-dose group	10	1.34 ± 0.23
Medium-dose group	10	1.68 ± 0.39^b^
High-dose group	10	1.82 ± 0.67^c^

Notes: blank group and model group were given normal saline 1 mL/100 g; the low-, medium-, and high-dose HAD-treated groups were given Heweianshen decoction of 5.25, 10.5, and 21 g·kg^−1^·d^−1^, respectively. Compared with the blank group, ^a^ *P* < 0.01; compared with the model group, ^b^ *P* < 0.05; compared with the model group, ^c^ *P* < 0.01.
